# Vaccine-Induced Protection of Rhesus Macaques against Plasma Viremia after Intradermal Infection with a European Lineage 1 Strain of West Nile Virus

**DOI:** 10.1371/journal.pone.0112568

**Published:** 2014-11-13

**Authors:** Babs E. Verstrepen, Herman Oostermeijer, Zahra Fagrouch, Melanie van Heteren, Henk Niphuis, Tom Haaksma, Ivanela Kondova, Willy M. Bogers, Marina de Filette, Niek Sanders, Linda Stertman, Sofia Magnusson, Orsolya Lőrincz, Julianna Lisziewicz, Luisa Barzon, Giorgio Palù, Michael S. Diamond, Stefan Chabierski, Sebastian Ulbert, Ernst J. Verschoor

**Affiliations:** 1 Department of Virology, Biomedical Primate Research Centre (BPRC), Rijswijk, The Netherlands; 2 Animal Science Department, Division of Pathology and Microbiology, BPRC Rijswijk, The Netherlands; 3 Laboratory of Gene Therapy, Faculty of Veterinary Sciences, Ghent University, Merelbeke, Belgium; 4 Novavax AB, Uppsala, Sweden; 5 eMMUNITY, Inc., Budapest, Hungary; 6 Department of Molecular Medicine, University of Padova, Padova, Italy; 7 Departments of Medicine, Molecular Microbiology and Pathology and Immunology, Washington University School of Medicine, St. Louis, Missouri, United States of America; 8 Department of Immunology, Fraunhofer Institute for Cell Therapy and Immunology, Leipzig, Germany; Metabiota, United States of America

## Abstract

The mosquito-borne West Nile virus (WNV) causes human and animal disease with outbreaks in several parts of the world including North America, the Mediterranean countries, Central and East Europe, the Middle East, and Africa. Particularly in elderly people and individuals with an impaired immune system, infection with WNV can progress into a serious neuroinvasive disease. Currently, no treatment or vaccine is available to protect humans against infection or disease. The goal of this study was to develop a WNV-vaccine that is safe to use in these high-risk human target populations. We performed a vaccine efficacy study in non-human primates using the contemporary, pathogenic European WNV genotype 1a challenge strain, WNV-Ita09. Two vaccine strategies were evaluated in rhesus macaques (*Macaca mulatta*) using recombinant soluble WNV envelope (E) ectodomain adjuvanted with Matrix-M, either with or without DNA priming. The DNA priming immunization was performed with WNV-DermaVir nanoparticles. Both vaccination strategies successfully induced humoral and cellular immune responses that completely protected the macaques against the development of viremia. In addition, the vaccine was well tolerated by all animals. Overall, The WNV E protein adjuvanted with Matrix-M is a promising vaccine candidate for a non-infectious WNV vaccine for use in humans, including at-risk populations.

## Background

West Nile virus (WNV) is a mosquito-borne flavivirus that is maintained in an enzootic transmission cycle between avian hosts and mosquito vectors, but WNV can also be transmitted to humans and other mammals [1]. Infection in humans is asymptomatic in most cases, but in about 20% of infections it presents as West Nile fever (WNF), and in less than 1% of cases, mainly in elderly and immunosuppressed individuals, as West Nile neuroinvasive disease (WNND) [1].

In recent years, WNV infection has become a public health concern in Europe because of the increasing number of human outbreaks with severe neurological consequences and mortality [2]–[7]. In addition, WNV has continued to cause large epidemics in North America, such as those that occurred in Dallas, Texas, in 2012 [8].

Seven different phylogenetic lineages of WNV have been described so far [9], [10], but only WNV lineages 1 and 2 have been associated with disease in humans. The different WNV lineages are genetically related, and show 75% to 95% nucleotide identity. In particular, WNV lineage 1 and lineage 2 viruses demonstrate about 75% nucleotide identity and 94% amino acid sequence identity [10], [11]. Lineage 1 has a worldwide geographic distribution, and in Europe lineage 1 viruses have been responsible for human cases of WNND in the Mediterranean countries and Eastern Europe since the 1950s [10]. Lineage 2 viruses were originally found only in sub-Saharan Africa and Madagascar, but in 2004 this lineage emerged in Europe, and has spread across the continent [12]. In 1999, a highly virulent WNV lineage 1 strain was introduced into the United States, and rapidly became endemic throughout the continent, affecting wild birds and mammals [13]. Moreover, this strain named NY99, caused a high number of cases of WNF and WNND, leading to considerable morbidity and mortality in humans.

The increasing number of outbreaks, as well as emergence of novel strains belonging to both major lineages, emphasizes the necessity to develop a WNV vaccine [4], [5], [14], [15]. Several WNV vaccines have been licensed for use in horses, but no vaccine for human use has been approved yet [16]. A number of WNV vaccine candidates are currently at different stages of development, and make use of recombinant proteins, plasmid DNA vectors, or chimeric live-attenuated virus approaches [17]. The majority of these vaccines are based on the WNV envelope (E) protein. Either E protein in its native form, a truncated subunit protein 80E, the WNV E immunodominant domain III, or combinations of these compounds are used as immunogens. Most vaccine candidates have been evaluated in rodents [18]–[21], but such studies may have limited prognostic value for its efficacy in humans given the significant differences in B and T-cell repertoire between both species.

Because of their genetic relatedness to humans, and their relative susceptibility to WNV infection, rhesus macaques may provide a better animal model for the evaluation of the immunogenicity and efficacy of prototype human WNV vaccines. Candidate WNV vaccines that have been tested in non-human primates include recombinant chimeric yellow fever virus or dengue virus as backbone expressing WNV structural genes [22]–[27], or adjuvanted recombinant E protein [24]. Because of the high impact on human health after its introduction in North America, all WNV vaccines that have been tested in nonhuman primates were based on WNV-NY99, and no data are available of vaccine efficacy to more distantly related European WNV isolates.

We recently performed an experimental infection study in rhesus macaques and common marmosets using the European WNV genotype 1a strain, WNV-Ita09 [28]. Infection in rhesus macaques resulted in a transient viremia with a peak viral load at 2–3 days post-infection, and the emergence of IgM and IgG antibodies within 15 days of infection. After clearance of the viremic phase, WNV was still detectable in tissues like spleen, axillary and inguinal lymph nodes, which resembles the situation observed in human infections [29]. Therefore, rhesus macaques were used in this study to assess vaccine efficacy against the European WNV-Ita09 strain.

Neutralizing antibodies are associated with protection against WNV infection [30], [31], whereas T-cells contribute to clearance of infection [32], [33]. Because the E protein of WNV is a primary target for CD8 T-cells [34] and neutralizing antibodies [35], we selected it for use in a human WNV subunit vaccine. The immunogens used in our study were derived from the WNV-NY99 strain, and were either the ectodomain of the WNV E protein that was expressed in *E. coli* [36], or a DNA vector expressing the WNV E ectodomain [37].

To increase vaccine induced T-cell responses we formulated the E protein in Matrix-M (Novavax AB). The adjuvant Matrix-M is composed of a specific purified saponin fraction obtained from the tree *Quillaja saponaria* Molina, phosphatidyl choline and cholesterol, and has been shown to increase the migration of the antigen towards the draining lymph nodes [38], [39]. An additional strategy to boost the T-cell responses is to prime the immune system with a DNA vaccine [40]. Here, we used a DNA vector expressing the WNV E protein in combination with a mannose-conjugated linear polyethylenimine delivery reagent; WNV-DermaVir [41], [42]. The mannose ligand enhances the delivery of DNA to cells expressing mannose-receptors, such as macrophages and dendritic cells, and thus, promotes antigen presentation to T-cells [43].

Two different WNV vaccine strategies were evaluated for immunogenicity and efficacy against WNV-Ita09 challenge. The first strategy consisted of three immunizations with recombinant E protein adjuvanted with Matrix-M. The second strategy entailed a priming immunization with WNV-DermaVir, followed by two booster immunizations with recombinant E protein and Matrix-M. Nine weeks after the last immunization the animals were challenged with the European WNV-Ita09 strain. Both strategies had been evaluated previously in mice, and in that model induced neutralizing antibodies and WNV-specific cellular immune responses [42], [44]. Here, in macaques, we observed robust humoral and cellular responses in both vaccination groups although the responses were higher in the protein-only immunization group. Animals in both groups showed consistent vaccine-induced IFNγ responses prior to WNV exposure. After challenge, all vaccinated macaques were completely protected against the development of viremia.

## Methods

### Ethics statement

This protocol was approved by the Institutional Animal Care and Use Committee (BPRC Dier Experimenten Commissie, BPRC-DEC; DEC advice #724). The qualification of the members of this committee, including their independence from a research institute, is requested in the Dutch law on animal Experiments (Wet op de Dierproeven, 1996). At the BPRC, all animal handling is performed within the Department of Animal Science (ASD) according to Dutch law. A large experienced staff is available, including full-time veterinarians and a pathologist. ASD is regularly inspected by the responsible authority (Voedsel en Waren Autoriteit, VWA), and by an independent Animal Welfare Officer.

The Council of the Association for Assessment and Accreditation of Laboratory Animal Care (AAALAC International) has awarded full accreditation to the BPRC. The BPRC is fully compliant with international demands on animal studies and welfare as set out by the European Convention for the Protection of Vertebrate Animals used for Experimental and other Scientific Purposes, Council of Europe (ETS 123 including the revised Appendix A), Dutch implementing legislation, and the Guide for Care and Use of Laboratory Animals.

The rhesus macaques (*Macaca mulatta*) used in this study were captive-bred for research purposes and housed socially at the Biomedical Primate Research Centre (BPRC) in Rijswijk, The Netherlands. BPRC facilities comply with Dutch law on animal experiments (Wet op de Dierproeven, and its adaptations as published in the Staatscourant), the European Council Directive 86/609/EEC, as well as with the ‘Standard for humane care and use of Laboratory Animals by Foreign institutions’ identification number A5539-01, provided by the Department of Health and Human Services of the United States of America’s National Institutes of Health (NIH).

During the experiment, the animals were pair-housed in a BSL3-facility with spacious cages and were provided with commercial food pellets supplemented with appropriate treats. Drinking water was provided *ad libitum.* Enrichment was provided in the form of pieces of wood, mirrors, food puzzles, a variety of other home-made or commercially available enrichment products. Animals were monitored daily for health and discomfort.

All steps were taken to ameliorate the welfare and to avoid any suffering of the animals. All experimental interventions (immunizations, intradermal injection of WNV, blood samplings) were performed under anesthesia using ketamine. Before euthanasia, animals were first sedated deeply with ketamine, and subsequently euthanized by intracardiac injection of an overdose of pentobarbital.

### Animals

Eighteen rhesus macaques (*Macaca mulatta*) were used in this study. All monkeys were adult animals, ranging in age from 5 to 12 years. The animals were in good physical health with normal baseline biochemical and hematological values. At the start of the study, the animals tested negative for antibodies to WNV. To prevent sex, age and weight bias, the animals were assigned randomly to different treatment groups.

### Vaccines

The ectodomain of the E protein (amino acid residues 1 to 404) of WNV-NY99 was cloned into the bacterial expression plasmid the pET21a, expressed in *E. coli* and purified as described previously [36]. This antigen was formulated Matrix-M, a mixture of 40 nm particles formed by two separate saponin fractions, i.e. Matrix-A and Matrix-C (Novavax AB, Uppsala, Sweden) [38]. WNV-DermaVir nanoparticles, containing a WNV DNA vaccine that expresses the ectodomain of WNV E protein, were prepared as previously described [42], [45].

### Experimental set up

**Figure 1:**
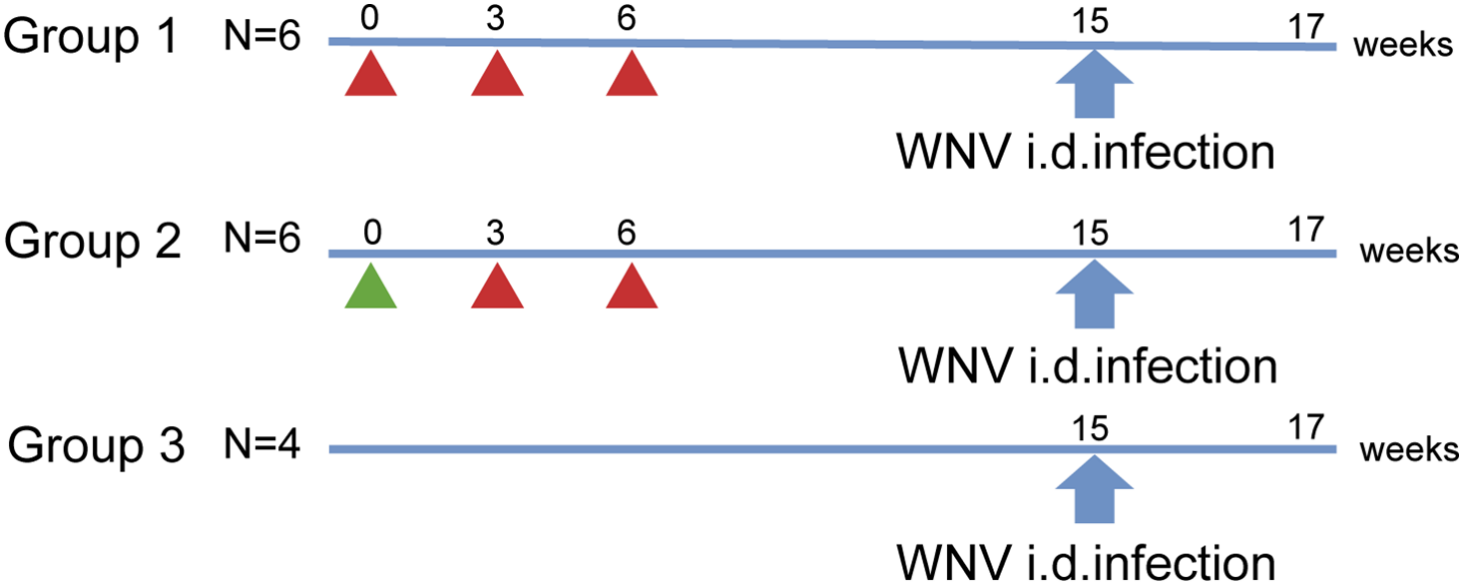
Study outline. Schematic representation of the study with two West Nile virus vaccine strategies. Group 1 received three immunizations with recombinant E protein adjuvanted with Matrix-M (red triangles) at indicated study weeks. Group 2 received one immunization of WNV-DermaVir (green triangle), followed by two immunizations with recombinant E protein adjuvanted with Matrix-M (red triangles). Nine weeks after the last immunization, all animals (including controls) were challenged intradermally with 2×10^5^ TCID_50_ of WNV-Ita09. All animals were euthanized 14 days post-challenge (study week 17).

A schematic outline of the study is given in **Figure 1**. The animals in group 1 were immunized via three consecutive intramuscular (IM) injections of 20 µg WNV-E mixed with 25 µg Matrix-M at weeks 0, 3 and 6. The animals in group 2 received 100 µg WNV-DermaVir at week 0, given as 8 intradermal injections of 100 µl each in the upper back. Subsequently, the animals were boosted twice at weeks 3 and 6 with 20 µg WNV-E mixed with 25 µg Matrix-M. Nine weeks after the last immunization, all animals, including those in the infection control group (group 3), were challenged by an intradermal injection in the upper back of 2×10^5^ TCID_50_ of WNV lineage 1a strain Ita09 [46] in 100 µl saline. This dose was found previously to productively infect rhesus macaques [28]. After WNV infection, the animals were observed daily for general condition, appetite, and stool until the end of the study, i.e. 14 days post-challenge.

During the immunization period, blood was collected using standard aseptic methods from the femoral vein at the start of the study, two weeks after each immunization, 5 weeks after the last booster immunization, and at challenge (week 15) for determination of biochemical and hematological parameters, and for the analysis of vaccine-induced humoral and cellular immune responses. After challenge, 0.5 ml blood samples were collected on a daily basis until euthanasia for viral load determination using real-time RT-PCR. Additional, larger blood volumes were collected at days 3, 7, and 14 post challenge for hematological and biochemical analysis, and for the evaluation of WNV-specific humoral and cellular immune responses.

### Biochemistry and hematology

A panel of hematological parameters, i.e. white blood cell count (WBC), red blood cell count (RBC), hemoglobin, hematocrit, mean cellular volume (MCV), mean corpuscular hemoglobin (MCH), platelets, neutrophils, lymphocytes, monocytes, eosinophils and basophils, was analyzed in peripheral blood using a Sysmex XT-2000iV Automated Hematology Analyzer (Sysmex Nederland B.V., Etten-Leur, The Netherlands). Biochemical analysis, i.e. creatinine, urea, bilirubin, gamma-glutamyltransferase (γGT), aspartate aminotransferase (AST), alanine aminotransferase (ALT), alkaline phosphatase, lactate dehydrogenase (LDH), iron, albumin, total protein, cholesterol and glucose, was assessed using a COBAS Integra 400 plus system (Roche Diagnostics Nederland B.V., Almere, The Netherlands).

### Characterization of humoral immune responses

WNV-specific antibodies in EDTA-plasma were detected by ELISA. Briefly, 96-well microtiter plates were coated overnight with 400 ng of the ectodomain of the WNV-NY99 E protein [36], or with 500 ng of hydrogen-peroxide-inactivated WNV-NY99 [47]. The coated plates were incubated for 2 h with 1∶50 diluted EDTA plasma, followed by 1 hr incubation with HRP-conjugated goat-anti-human IgG (Thermo Fisher Scientific, Schwerte, Germany). After washing, TMB-substrate (BioLegend, Fell, Germany) was added to the wells and the plate was incubated for 30 min at room temperature in the dark. Then, 1 M H_2_SO_4_ was added to stop the reaction and plates were measured at 450 nm and 520 nm (reference wavelength) in an ELISA Reader (Infiniti M200, Tecan, The Netherlands).

To determine the *in vitro* neutralizing capacity of sera from vaccinated macaques, plaque-reduction neutralization tests (PRNT50) were performed essentially as described in the Guidelines for plaque reduction neutralization testing of human antibodies to dengue virus (World Health Organization, 2007). Heat-inactivated EDTA plasma samples taken at various time points were serially diluted and mixed with 25 TCID_50_ of WNV-Ita09 (lineage 1), or WNV-AUT08 (lineage 2), before addition to adherent Vero cells. Cytopathic effect (CPE) was visualized using a standard microscope, and the TCID_50_ was calculated using the Karber formula [48].

### Determination of cell-mediated immune responses

Cell-mediated immune responses were determined in peripheral blood mononuclear cells (PBMCs) isolated from EDTA-treated blood. PBMCs were tested for WNV-specific secretion using WNV-E protein in ELIspot assays according to the manufacturers’ guidelines (U-CyTech, Utrecht, The Netherlands). ELIspot assays were performed on freshly isolated cells at weeks 0, 2, 5, 8, and 11.

To obtain more detailed information on the quality of WNV-specific responses, intracellular cytokine staining (ICS) was performed. Frozen PBMCs, isolated at weeks 0 and 15 were thawed, and intracellular staining was performed as described previously [49] using a panel of monoclonal antibodies; LIVE/DEAD-Aqua (Life Technologies, Grand Island, NY), CD3-AF700, CD8-V500, CD4-PE-Cy7 and IFNγ-PE (all Becton, Dickinson B.V., Breda, The Netherlands). Fluorescence was measured using a FACS LSR2 (Becton, Dickinson and Company, Breda, The Netherlands). Data were analyzed with FlowJo software, version 9.6.4 (Tree Star, Stanford University, USA).

### Virus detection in blood and tissue

Viral loads were determined in EDTA-plasma by quantitative real-time RT-PCR as previously described [28], [50]. To determine the presence of WNV in tissue samples, 1 mg of snap frozen tissue was added to 1 ml RPMI and was dissociated using a gentleMACS dissociator (Miltenyi Biotec B.V., Leiden, The Netherlands). The homogenate was centrifuged for 10 min at 820× g at room temperature, and the supernatant was filtered through a 40 µm filter. Viral RNA was isolated from 140 µl of filtered homogenate with the QIAamp Viral RNA Mini Kit (QIAGEN Benelux BV, Venlo, the Netherlands), and was subsequently analyzed by PCR, as described previously [28].

### Necropsy

Monkeys were euthanized by infusion of pentobarbital (Apharma, Duiven, The Netherlands), and full necropsy was performed. Based on the dissemination data of WNV obtained from an earlier experimental infection study [28], samples were collected from the following organs for PCR analysis; axillary lymph nodes (ln), inguinal ln, mesenteric ln, spleen, urinary bladder, kidney, cerebellum and hippocampus. All samples were snap frozen for WNV-RNA determination.

### Statistical analysis

Data obtained with the two vaccine strategies were analyzed and compared using an unpaired t-test in GraphPad Prism version 6.0.

## Results

### Induction of antibody responses against WNV by both vaccine strategies

Both vaccines were well tolerated by the animals and no local reactions were observed after immunization.

**Figure 2:**
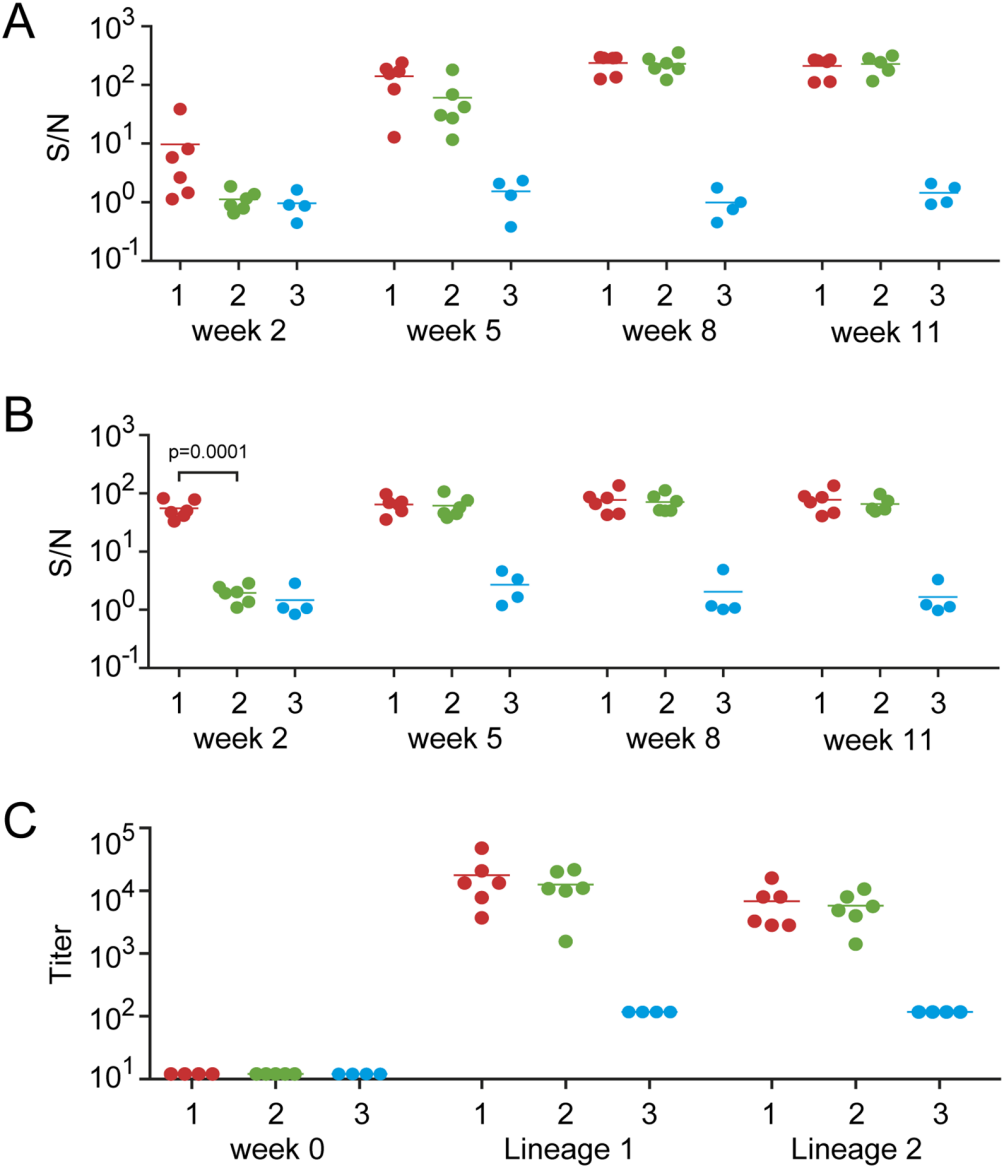
Vaccine-induced antibody responses. Antibodies reactive against (A) the ecto-domain of the WNV E protein, and (B) inactivated WNV were measured in the individual animals at indicated time points. Humoral responses were quantified as sample:negative ratio (S/N). Vaccine-induced neutralizing capacity (PRNT50) of macaque sera was determined using the plaque reduction neutralization test (C). Individual animals are depicted as dots: group 1 (red), group 2 (green), and group 3 (blue). The median value is indicated for each group. Unpaired t-test was used to compare the responses between the groups. Statistical significant differences were defined as p<0.05 and are indicated with arches in the figure.

Induction of WNV-specific IgG was measured two weeks after each immunization, and 4 weeks before WNV challenge (**Figure 1**). After the first immunization, very low levels of anti-WNV E IgG were detected (sample:negative ratio (S/N) <40) in only one macaque of the protein-only group (group 1) (**Figure 2A**). After the second protein immunization, 5 of 6 animals from group 1 showed high IgG titers directed against the ectodomain of E, although one animal failed to develop a response that exceeded background levels. In all group 1 animals the IgG response was boosted after the third protein immunization, and remained stable until at least week 11 (one animal was not tested at this time point). In animals of group 2, which first received a DNA vaccine prime followed by two protein booster immunizations, no detectable antibody responses were observed at two weeks after the DNA immunization. After the first E protein boost the levels of E-specific IgG were significantly higher in comparison to the group 1 animals after a single protein immunization, which indicates a priming effect of the DNA vaccine. After the second protein boost the antibody titers in DNA-protein group reached levels that were comparable to those measured in the protein-only group. As expected, no E-specific IgG levels were detected in the control animals prior to WNV challenge. The specificity of the WNV-E protein-specific IgG responses was confirmed by using inactivated whole virus as capture antigen in ELISA (**Figure 2B**).

Vaccine-induced neutralizing antibodies (VN) against WNV were measured using a plaque reduction assay on Vero cells in plasma samples collected at week 0 and week 11. No VN were detectable at the start of the immunization period, but plasma samples collected 5 weeks after the third immunization (week 11) inhibited the infectivity WNV-Ita09, with individual VN titers ranging from 1/3,698 to 1/48,000 (mean value 17,805) in group 1, and from 1/1,567 to 1/21,657 (mean value 12,534) in group 2 (**Figure 2C**). Vaccine-induced neutralizing antibodies cross-neutralized the lineage 2 WNV strain AUT08 with titers ranging from 1/2,828 to 1/160,00 (mean value 6,822) in group 1 animals, and ranging from 1/1,414 to 1/16,000 (mean value 5,771) in group 2 animals (**Figure 2C**). None of the animals in the control group showed neutralizing capacity against the lineage 1 or lineage 2 WNV isolates tested.

### CD8 T-cell responses in macaques elicited by WNV vaccines

**Figure 3:**
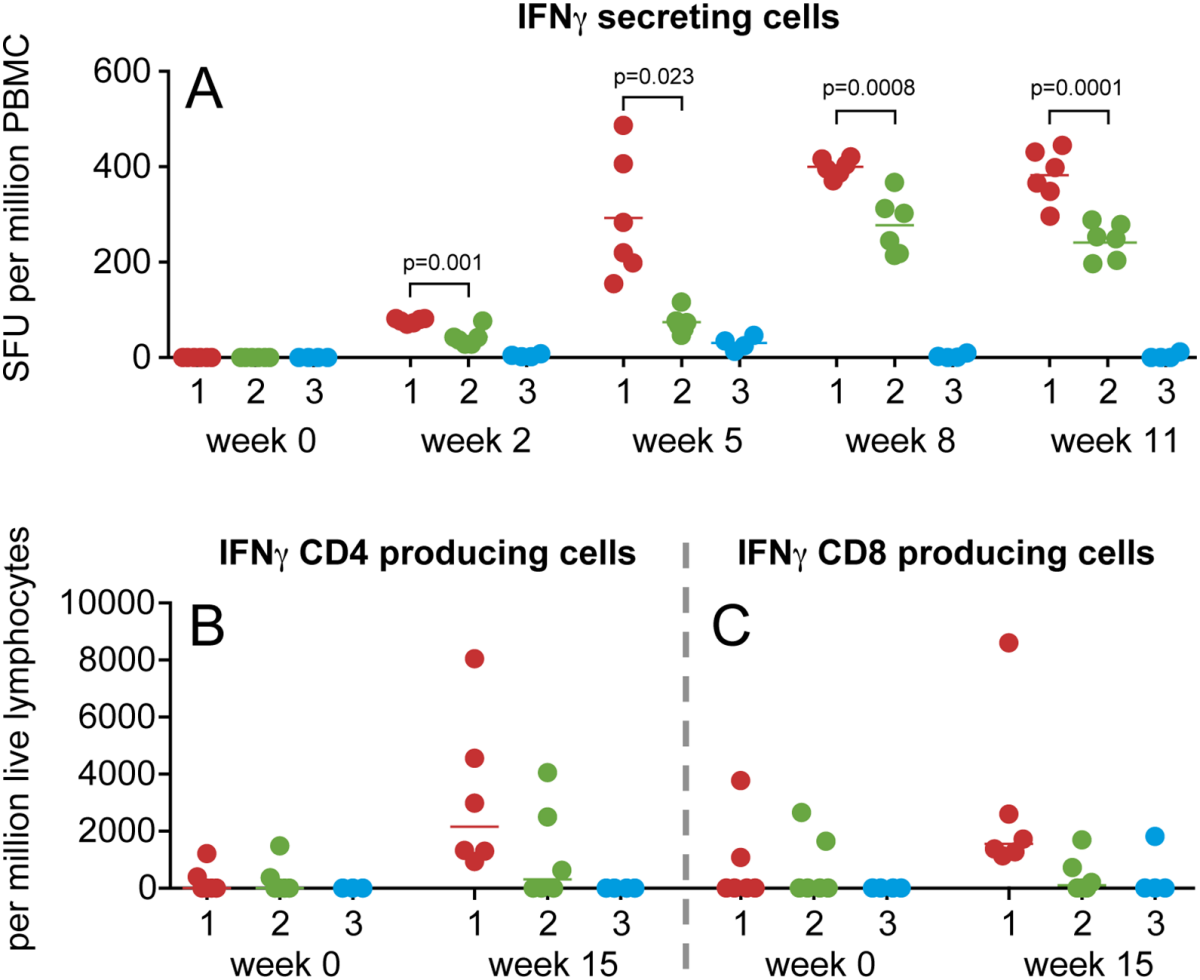
Vaccine-induced T-cell responses. A. IFNγ-secreting cells in blood of the individual animals measured in ELISpot. The responses are presented in spot-forming units (SFU) per million PBMCs. The WNV-specific T-cell responses were calculated by subtraction of the background responses (mean value of triplicate assays plus two times the standard deviation, minus medium alone). Intracellular staining of IFNγ produced by CD4 T-cells (panel B), and CD8 T-cells (panel C). Background IFNγ-responses (number of IFNγ-producing cells with medium alone) were subtracted. Individual animals are depicted as dots: group 1 (red), group 2 (green), and group 3 (blue). The median value is indicated for each group. Unpaired t-test was used to compare the responses between the groups. Statistical significant differences were defined as p<0.05.

To determine the cellular immunogenicity of the two WNV vaccine strategies, IFNγ ELIspot assays were performed on isolated peripheral blood mononuclear cells (PBMCs) (**Figure 3A**). Two weeks after the first immunization, a significant difference was observed between the number of spot-forming units (SFU) observed in PBMC from animals that received a protein immunization (70 to 82 SFU per million PBMC) or a DNA immunization (28 to 77 SFU per million PBMC) (p = 0.001). Two weeks after the second immunization, WNV-specific IFNγ responses in both groups were boosted to 155 to 487 SFU in macaques of group 1, and 47 to 117 SFU in the group 2 animals (p = 0.023). The final immunization further augmented the number IFNγ secreting cells in peripheral blood in both vaccine groups, with a more robust response in group 1 animals compared to group 2 (median values of 400 and 274 SFU, respectively, p = 0.0008). Three weeks later, minor changes were observed in the number of IFNγ-secreting cells in the different groups. Macaques of group 1 still showed significantly higher number of IFNγ-secreting cells compared to animals belonging to the DNA-protein group (p = 0.0001).

To determine if IFNγ was produced by CD4 T-cells, or by CD8 T-cells, ICS was performed on cells collected at weeks 0 and 15. Cells within the lymphocyte gate were selected based on the expression of CD3 (**Figure S1**). Next, CD4 T-cells and CD8 T-cells were analyzed for their intracellular expression of IFNγ. At week 15, the day of WNV challenge, IFNγ was produced by both CD4 (**Figure 3B**), and CD8 (**Figure 3C**) T-cells in all animals of group 1. This was not observed in the macaques that received the WNV-DermaVir priming immunization, followed by two protein immunizations (group 2). In this group, 2 of 6 animals had IFNγ-producing CD4 T-cells, and only one animal had IFNγ producing CD8 T-cells.

### Determination of WNV vaccine efficacy in rhesus macaques

At week 15, all of the animals were challenged intradermally with 2×10^5^ TCID_50_ of WNV lineage 1a strain Ita09. During the 14-day observation period, none of the macaques showed any behavioral changes or health complications. In addition, no changes in rectal body temperature (**Figure S2**), hematological and biochemical parameters were seen, suggesting that all animals remained clinically healthy during the 2-weeks post-challenge follow-up.

The protective capacity of both WNV vaccine strategies was assessed by measuring WNV RNA levels in plasma from the macaques by real-time RT-PCR, and in solid tissues by diagnostic PCR. All vaccinated macaques remained negative for WNV in plasma during the entire follow-up period. In contrast, one day after intradermal infection with WNV, 4 of 6 non-vaccinated controls had become positive for WNV (7.700 to 58.000 RNA copies/ml plasma) (**Table 1**). Two days post-challenge, 5 of 6 control animals were positive for WNV RNA. By day 6 after infection, none of macaques had measurable levels of WNV RNA in peripheral blood. In animal R08058, one of the macaques in the non-vaccinated control group, viral RNA was not detected in peripheral blood at any of the time points tested.

**Table 1:** Detection of West Nile virus load in plasma of vaccinated and control rhesus macaques.

	Days post-exposure										
	0	1	2	3	4	5	6	8	10	12	14
**Group 1: protein only**											
R08032	−	−	−	−	−	−	−	−	−	−	−
R08101	−	−	−	−	−	−	−	−	−	−	−
R06024	−	−	−	−	−	−	−	−	−	−	−
R06078	−	−	−	−	−	−	−	−	−	−	−
R03008	−	−	−	−	−	−	−	−	−	−	−
R01080	−	−	−	−	−	−	−	−	−	−	−
**Group 2: DNA prime, protein boost**											
R08106	−	−	−	−	−	−	−	−	−	−	−
R07121	−	−	−	−	−	−	−	−	−	−	−
R06111	−	−	−	−	−	−	−	−	−	−	−
R06070	−	−	−	−	−	−	−	−	−	−	−
R03020	−	−	−	−	−	−	−	−	−	−	−
R02073	−	−	−	−	−	−	−	−	−	−	−
**Group 3: control animals**											
R08058	−	−	−	−	−	−	−	−	−	−	−
R06047	−	−	2900	**18000**	1900	−	−	−	−	−	−
R03027	−	**58000**	43000	16800	19000	−	−	−	−	−	−
R02085	−	7700	2800	5500	**61000**	−	−	−	−	−	−
R01034	−	27000	770	**63000**	16000	−	−	−	−	−	−
R05066	−	46000	86000	**110000**	71000	194	−	−	−	−	−

Real time RT-PCR was used to quantify WNV RNA load in plasma at indicated time points after challenge. Virus loads are presented in copies per ml. Peak virus loads are given in bold.

In addition to quantifying WNV by real-time PCR, we tested the plasma taken at 1 to 5 days after WNV exposure for the presence of infectious WNV particles. Plasma of EDTA-treated blood samples was serially diluted, and cultured for 7 days on Vero cells. Only plasma from R03027 (a non-vaccinated control) collected 3 days after WNV exposure caused CPE in Vero cells. Based on a standard curve analysis, the virus titer in this sample was calculated 144 infectious particles per ml of plasma. Sequence analysis confirmed that the CPE in Vero cells was caused by WNV-Ita09 infection.

### WNV tissue distribution in vaccinated and non-vaccinated rhesus macaques

**Figure 4:**
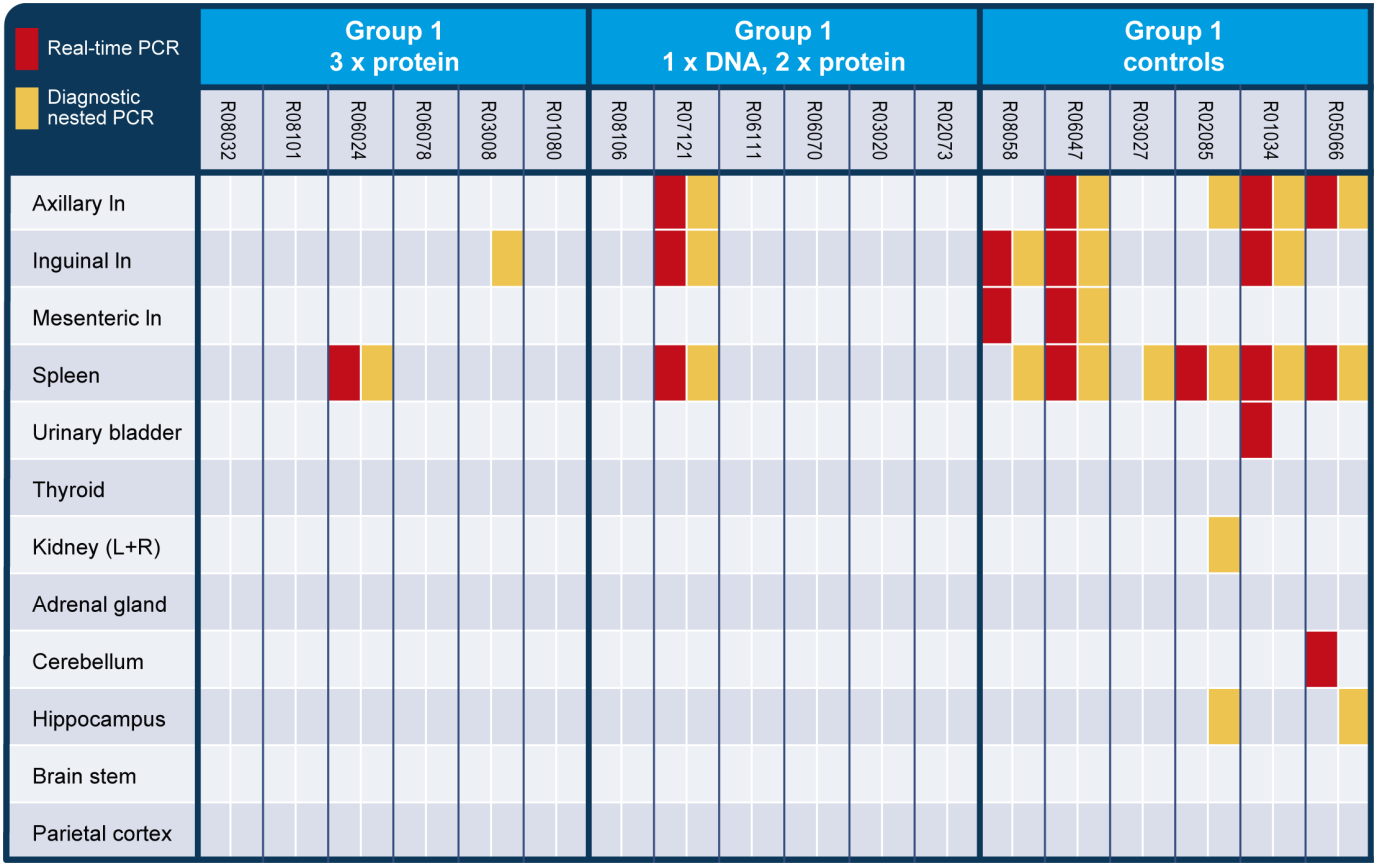
Detection of West Nile virus in tissue samples. Tissue samples were analyzed for the presence of WNV RNA by qualitative real-time PCR (red) or a nested PCR assay (orange).

In contrast to the control animals, the vaccinated animals did not show plasma viremia despite exposure to WNV. To assess if the vaccine strategies employed in this study conferred sterilizing immunity, we performed PCR analysis on solid tissues that were collected at euthanasia, 14 days post-challenge. **Figure 4** shows the data obtained by qualitative real-time PCR and a nested PCR assay performed on selected tissue samples. WNV RNA was detected in the peripheral lymph nodes (axillary, inguinal or mesenteric ln) from 5 out of 6 non-vaccinated controls. Notably, WNV RNA was also present in the peripheral lymph nodes of animal R08058, the only control that did not show WNV RNA in plasma. In the unvaccinated macaque R03027, although WNV RNA was present in plasma, we did not find WNV RNA in peripheral lymph nodes. In the spleen of this animal, however, the nested PCR assay did detect WNV RNA. The spleen tested positive for WNV RNA in all 6 control animals in at least one of the two PCR assays used. In contrast, WNV RNA was detected in the spleen of 2 out of 12 vaccinated rhesus macaques, including one animal from each of the groups (R06024 and R07121).

The kidney and urinary system have been suggested as potential target organs for WNV infection in humans [2], [51]. Here, only two control animals tested positive for WNV RNA in the urinary bladder (R01034) or kidney (R02085). Because WNV disease is associated with neuroinvasion, different parts of the brain [29], the cerebellum, the hippocampus, the brain stem, and the parietal cortex, were tested. WNV RNA was observed in cerebellum of control animal R05066, and in the hippocampus of 2 control monkeys, i.e. R02085 and R05066.

## Discussion

Since its introduction in 1999 into the USA, and the subsequent spread into the New World, WNV has emerged as a serious threat to public health. This opinion is confirmed by an increasing incidence of WNV infections in South-East Europe caused by lineage 1 and 2 WNV strains. Currently, no antiviral treatment or vaccine is available to protect humans from WNV infection. In our study, two WNV vaccine strategies (protein prime + protein boost and DNA prime + protein boost) fully protected monkeys against the development of viremia. Although sterilizing immunity was not achieved, only 3 of 12 vaccinated macaques were positive for WNV RNA in one or more solid tissues compared to 6 out of 6 challenge controls.

This project aimed to develop a vaccine against WNV that is safe for use in the high-risk human target populations, elderly individuals and those having a compromised immune system. Our prototype WNV vaccines were composed of antigens that are safe to use, as they are well-characterized recombinant components (proteins, DNA) that lack the ability to replicate. In preparation of the monkey study, the individual antigens, in combination with DermaVir and Matrix-M, showed safety, immunogenicity, and efficacy in rodents [42], [44].

Protein-based vaccines can efficiently protect against a number of viruses by eliciting antibodies, while priming the immune system with a DNA vaccine has been shown to induce CD8 T-cell-mediated immunity [40]. The correlate of protection against WNV infection has not been fully elucidated although CD8 T-cell and WNV-specific antibody responses are associated with protection from disease or infection [31], [32]. The ectodomain of the E protein is highly immunogenic and contains multiple CD8 and CD4 T-cell epitopes [52]–[54], and was consequently the immunogen of choice in our study. The combination of WNV-E adjuvanted with Matrix-M potently induced WNV-specific IgG antibodies in the macaques, even after the first immunization. In contrast, anti-WNV IgG was not observed after the priming immunization with the WNV-DermaVir nanoparticles. However, when WNV-DermaVir was combined with protein/Matrix-M booster immunizations, a priming effect on the humoral immune responses was seen. In mice, De Filette *et al*. [42] also demonstrated that WNV-DermaVir immunization failed to induce a measurable humoral immune response by itself, but upon protein boosting, DNA-vaccinated mice showed a marked increase in IgG and neutralizing antibody titers against WNV. In addition, mice that were given a WNV-DermaVir priming, followed by a protein boost had a higher amount of IL-4 and IFNγ-producing cells than mice that were given protein immunizations alone. This contrasts with our findings in the rhesus macaques. No evidence was found for an improved CD8 T-cell response due to the WNV-DermaVir priming. However, though the numbers were modest, a CD8 T-cell response was induced in the animals that received the WNV-DermaVir prime while a clear IFNγ producing CD8 T-cell population was shown in all animals from group 1 prior to the challenge. It is conceivable that the limited effect of the DNA priming immunization was caused by use of a too low dose. Others, using 5 to 10 times more DNA than the 100 µg DNA used in this study, did detect a strong priming effect on the immune system of rhesus macaques that resulted in higher and broader T-cell responses [55], [56].

We observed cross-protection between North American and European lineage 1 strains, as the vaccine components were based on WNV-NY99, and the challenge strain used in the monkeys was WNV-Ita09 (>99% sequence identity). In mice, E protein/Matrix-M immunizations also afforded protection against lethal challenge with a lineage 2 strain of WNV [44]. This was not evaluated *in vivo* in macaques, but *in vitro* assays for detection of virus neutralizing IgG showed cross-neutralization of the lineage 2 WNV-AUT08 strain. This is likely because of the conservation of dominant neutralizing epitopes in different regions of WNV E protein between lineage 1 and 2 strains [57].

At present, our results do not tell us which WNV vaccination strategy is best. Both induced complete protection against viremia, but failed to induce sterilizing immunity against intradermal challenge. Although it is speculative, the vaccine may have failed to achieve sterilizing immunity because of the relatively high challenge dose used. Depending on the mosquito species, the dose of WNV inoculated by one mosquito during blood feeding varies between 10^4^ and 10^5^ PFU [58], and thus the 2.10^5^ TCID_50_ challenge dose may have been relatively high.

Several WNV vaccines have been clinically evaluated [59]. Chimeric virus approaches, based of the yellow fever vaccine strain YFV-17D or using dengue virus as viral backbone, showed good immunogenicity in healthy volunteers, but may be unacceptable for vaccine-licensing because of the risk of residual pathogenicity, or reversal to pathogenicity in the human immune-compromised target populations. Safer vaccines that were based on naked DNA or protein subunits also showed good immunogenicity and induction of neutralizing antibodies in clinical phase I trials, and may therefore be better alternatives. In our pre-clinical macaque model we used a similar subunit and/or DNA vaccine approach, but instead used a European WNV challenge virus. Both the protein only and DNA-protein immunization strategies induced strong humoral and cellular immune responses, and protected healthy rhesus macaques from WNV infection. In recent years, human cases on WNF and WNND in Europe were also caused by WNV lineage 2 viruses, viruses previously thought to be less pathogenic to humans [60]. It is thus of major importance that our vaccines also elicited neutralizing antibodies that cross-reacted *in vitro* with a WNV lineage 2 strain. It can therefore be concluded that the vaccines described here are promising candidates for the further development of WNV vaccines for at-risk human populations.

## Supporting Information

Figure S1 **Gating strategy for intracellular IFNγ staining in CD4 and CD8 T-cells.** Representative gating strategy to define intracellular IFNγ-staining in CD4 and CD8+ T-cells of vaccinated rhesus macaques. Cytokine-producing T-cells were defined as LIVE/DEAD negative and CD3 positive cells. Next, CD4 positive cells and CD8 positive T-cells were analyzed for IFNγ production.
(TIF)

Figure S2 **Rectal body temperatures of rhesus monkeys during the immunization period and after WNV challenge.** Rectal body temperature (°C) measured at indicated time points in animals from group 1 (panel A; red), group 2 (panel B; green), and group 3 (panel C; blue). Median rectal body temperature (D) per group at indicated days after experimental WNV infection. Statistically significant differences were defined as p<0.05 and are indicated with arches in the figure.
(TIF)
