# Supplementary figures and images for: Vaccine-Induced Protection of Rhesus Macaques against Plasma Viremia after Intradermal Infection with a European Lineage 1 Strain of West Nile Virus
Source: PLoS One. 2014 Nov 13;9(11):e112568. doi: 10.1371/journal.pone.0112568 (PMC4231036; doi:10.1371/journal.pone.0112568)

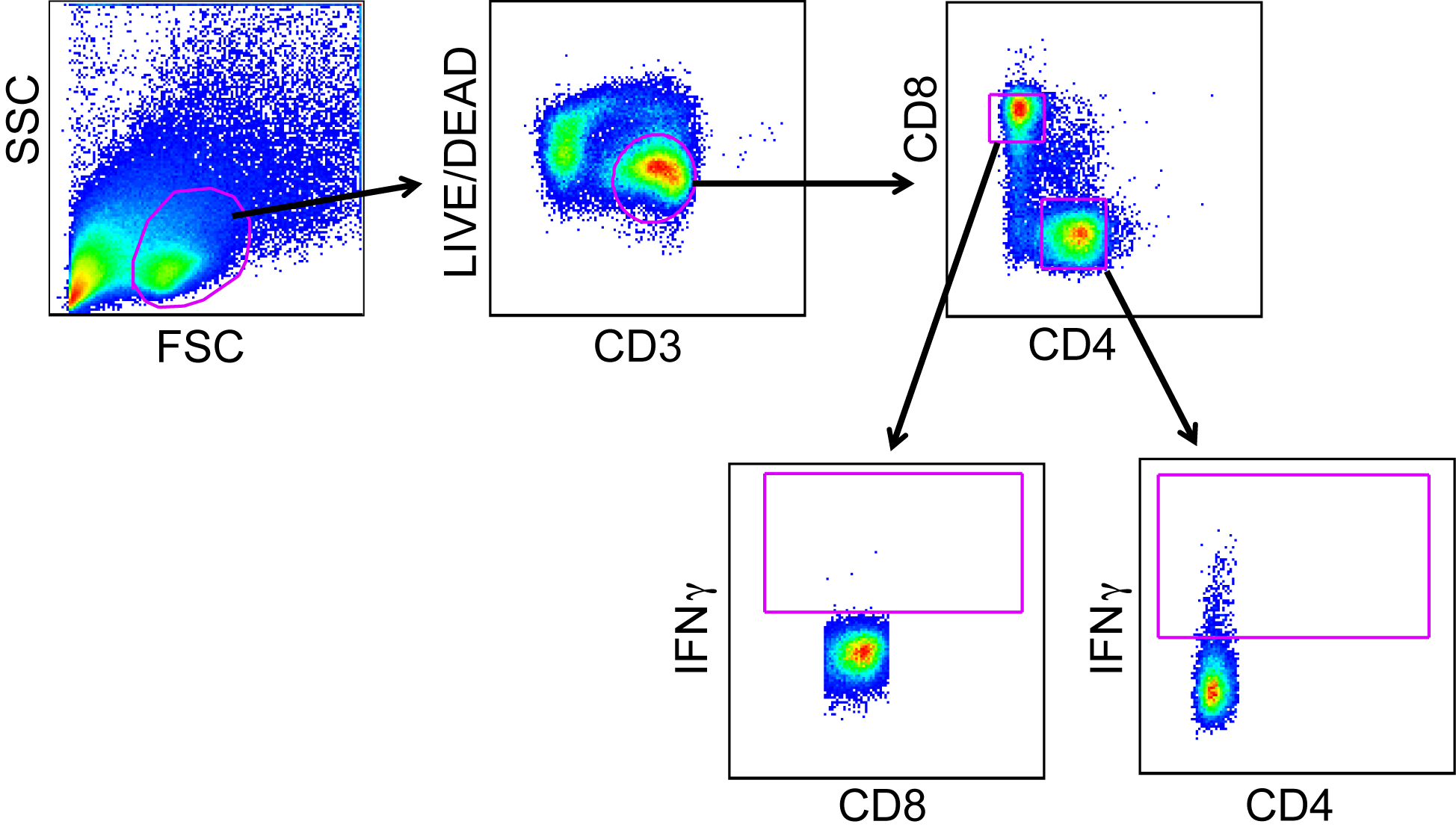

Supplement: Figure S1 — Gating strategy for intracellular IFNγ staining in CD4 and CD8 T-cells. Representative gating strategy to define intracellular IFNγ-staining in CD4 and CD8+ T-cells of vaccinated rhesus macaques. Cytokine-producing T-cells were defined as LIVE/DEAD negative and CD3 positive cells. Next, CD4 positive cells and CD8 positive T-cells were analyzed for IFNγ production. (TIF) [file pone.0112568.s001.tif]

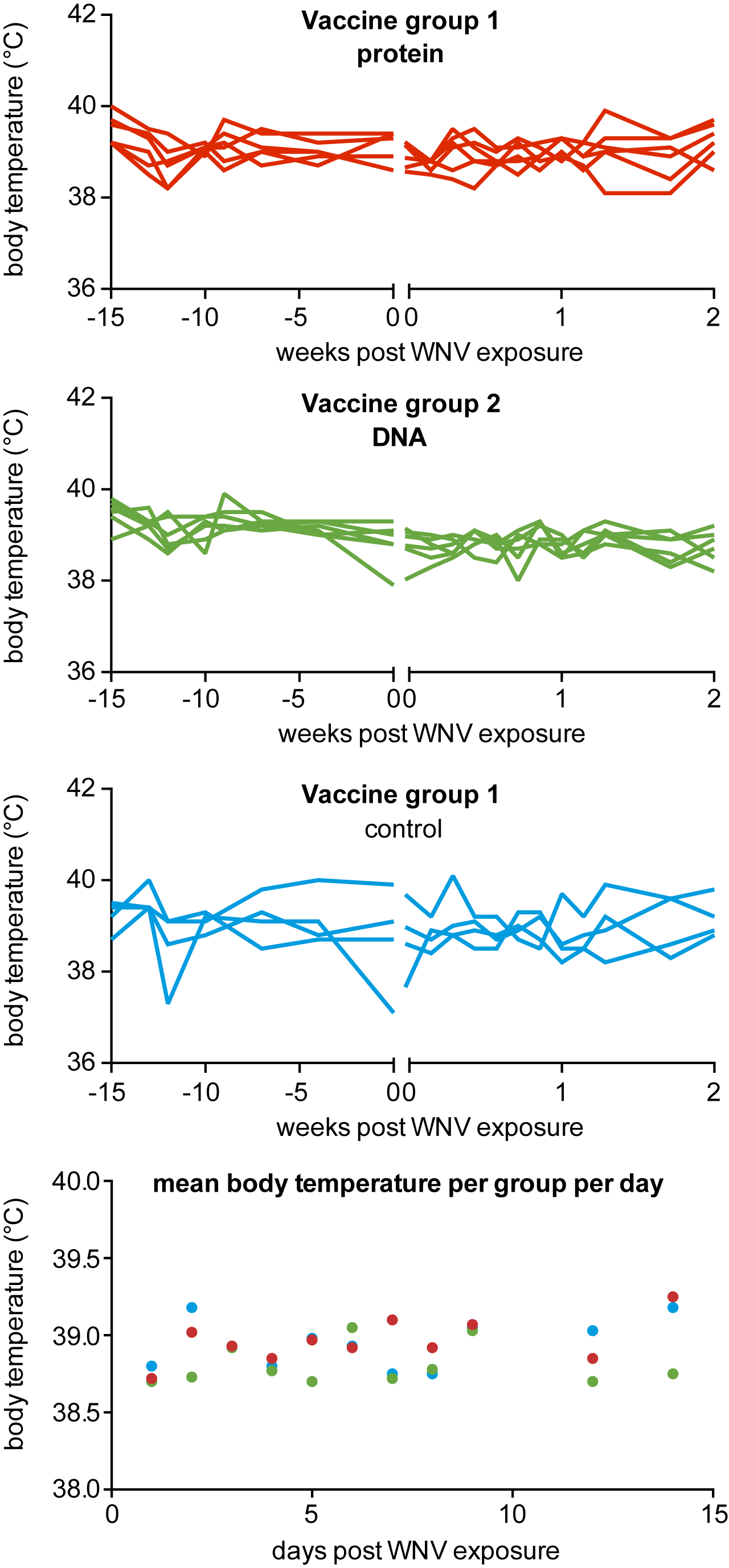

Supplement: Figure S2 — Rectal body temperatures of rhesus monkeys during the immunization period and after WNV challenge. Rectal body temperature (°C) measured at indicated time points in animals from group 1 (panel A; red), group 2 (panel B; green), and group 3 (panel C; blue). Median rectal body temperature (D) per group at indicated days after experimental WNV infection. Statistically significant differences were defined as p<0.05 and are indicated with arches in the figure. (TIF) [file pone.0112568.s002.tif]
